# Antimicrobial susceptibility of gram-negative bacilli isolated from intra-abdominal and urinary-tract infections in Mexico from 2009 to 2015: Results from the Study for Monitoring Antimicrobial Resistance Trends (SMART)

**DOI:** 10.1371/journal.pone.0198621

**Published:** 2018-06-21

**Authors:** Alfredo Ponce-de-Leon, Eduardo Rodríguez-Noriega, Rayo Morfín-Otero, Dora P. Cornejo-Juárez, Juan C. Tinoco, Areli Martínez-Gamboa, Carmen J. Gaona-Tapia, M. Lourdes Guerrero-Almeida, Alexandra Martin-Onraët, José Luis Vallejo Cervantes, José Sifuentes-Osornio

**Affiliations:** 1 Clinical Microbiology Laboratory, Salvador Zubirán National Institute of Medical Sciences and Nutrition, Mexico City, Mexico; 2 Department of Infectious Diseases, Salvador Zubirán National Institute of Medical Sciences and Nutrition, Mexico City, Mexico; 3 Hospital Civil de Guadalajara, Fray Antonio Alcalde, Guadalajara, Jalisco, Mexico; 4 Instituto de Patología Infecciosa y Experimental, Centro Universitario Ciencias de la Salud, Universidad de Guadalajara, Guadalajara, Jalisco, Mexico; 5 Department de Infectious Diseases, National Cancer Institute, Mexico City, Mexico; 6 Hospital General de Durango, Durango, Mexico; 7 MSD de México, Mexico City, Mexico; Centers for Disease Control and Prevention, UNITED STATES

## Abstract

Antimicrobial resistance is an increasing worldwide concern, which poses unique challenges for the effective prevention and treatment of several infections, especially the ones triggered by organisms producing extended-spectrum β-lactamases (ESBL). Here, we present the surveillance results of the Study for Monitoring Antimicrobial Resistance Trends (SMART) of Gram-negative bacilli isolated from intra-abdominal infections (IAI, n = 1,235) and urinary-tract infections (UTI, n = 2,682), collected in Mexico from 2009 to 2015. Susceptibility and ESBL status were determined according to the Clinical and Laboratory Standards Institute (CLSI) broth microdilution method. Both *E*. *coli* (57%) and *K*. *pneumoniae* (12%) were the most frequently reported organisms, as well as the ones with the highest prevalence of ESBL-producing isolates (54% and 39%, respectively). The overall prevalence of ESBL-producing organisms was higher in nosocomial infections than in community-acquired infections (21% *vs*. 27%). The ESBL rates were 36% for IAI (953/2,682) and 37% for UTI (461/1,235). In addition, ertapenem, imipenem and amikacin were the antibiotics that mostly preserved bacterial susceptibility. Our results show consistency with global trends, although higher than the rates observed in Latin America.

## Introduction

Antimicrobial resistance is an increasing worldwide concern, which poses unique challenges for microbiologists and infectious disease specialists regarding the effective prevention and treatment of several infections. This is especially alarming when considering the organisms producing extended-spectrum β-lactamases (ESBL). These enzymes are rapidly adaptable and able to inhibit the action of several antibiotics. They have the ability to hydrolyze most of the fluoroquinolones and β-lactam antibiotics, including penicillins, third-generation of cephalosporins, and the monobactam aztreonam [1–3]. Carbapenems are still the antimicrobial class of choice for the treatment of ESBL-producing organisms. However, in the last years, carbapenem-resistant Enterobacteriaceae (CRE) have also been reported, and widely spread worldwide, including Latin American countries [4–6]. This situation is exacerbated by the widespread misuse of antibiotics and has the consequence of limiting therapeutic options for various infections.

Urinary tract infections (UTI) and intra-abdominal infections (IAI) are among the most common infections, and are mainly caused by Gram-negative bacteria (GNB), in particular *Escherichia coli* and *Klebsiella* species [7]. Since the 1980s, ESBL-producing Enterobacteriaceae have been considered the major cause of nosocomial infections [3]. Ten years later, ESBL-producing *E*. *coli* also emerged as an etiological agent in the community-acquired UTIs [3, 8]. Tough most of the community-acquired infections caused by these organisms are UTIs, recent cases of IAI and associated bloodstream infections caused by ESBL-producing *E*. *coli* have been reported [8–10]. The enhanced widespread resistance of microorganisms causing nosocomial and community-acquired infections highlights the importance of knowing the antimicrobial community of each region and their susceptibility patterns. Surveillance programs were proven to be efficient tools to monitor antimicrobial resistance and to guide microbiologists and infectious disease specialists in optimizing treatment strategies [11].

The Study for Monitoring Antimicrobial Resistance Trends (SMART) [12–16] is a surveillance program implemented worldwide to monitor the *in vitro* susceptibility of clinical aerobic and facultative GNB isolates from UTI and IAI. Collection of isolates from IAI started in 2002, and from UTI started in late 2009. The main goals of the SMART study are to analyze the resistance trends of these isolates to ertapenem and 11 other selected antimicrobials, permitting longitudinal analyses to determine if susceptibility patterns change over time. In 2013, there were 187 sites worldwide participating in SMART, 4 of which located in Mexico. In this analysis, we present the latest results of UTI and IAI from the SMART study in Mexico from the surveillance period between 2009 and 2015. Additionally, the trends of antibiotic resistance of ESBL-producing bacteria and the type of infection (nosocomial or community-acquired infection) were also analyzed.

## Material and methods

### Isolate collections and study sites

All bacterial isolates were collected from intra-abdominal or urinary samples from adult, hospitalized patients of both sexes. Samples were analyzed prospectively in two General Hospitals (*Hospital General de Durango* and *Hospital Civil de Guadalajara*) and in two National Institutes of Health (*Instituto Nacional de Ciencias Médicas y Nutrición Salvador Zubirán* and *Instituto Nacional de Cancerología*) in Mexico, from 2009 to 2015. The collection of UTI isolates only started in 2010. To avoid duplicates, only one strain per species and per patient was included. The intra-abdominal samples were collected from surgical procedures that involved the abdominal cavity. The infections were categorized as community-acquired (isolates obtained in less than 48 hours of hospitalization) or nosocomial (isolates obtained after 48h of hospitalization), according to the standard criteria of the Centers for Disease Control and Prevention (CDC) [17].

All results were collected in an excel database and analyzed using descriptive statistics.

### Antimicrobial susceptibility

The identified isolates were sent to a central microbiology laboratory (International Health Management Associates, Inc., Schaumburg, Illinois, USA) for further species confirmation and antimicrobial susceptibility testing. Susceptibility and ESBL status were determined according to the Clinical and Laboratory Standards Institute (CLSI) broth microdilution method. Minimum inhibitory concentration (MIC) interpretive criteria followed the 2014 M100-S24 guidelines of the CLSI. The susceptibility of all Gram-negative isolates combined was calculated using breakpoints appropriate for each species and assuming a 0% susceptibility for species with no breakpoints for any given drug. The antimicrobial agents tested were the following: ertapenem (ETP), imipenem (IMP), Piperacillin-Tazobactam (TZP), Ampicillin-Sulbactam (SAM), cefoxitin (FOX), ceftazidime (CAZ), ceftriaxone (CRO), cefotaxime (CTX), cefepime (FEP), levofloxacin (LVX), ciprofloxacin (CIP) and amikacin (AMK). In the case of susceptibility of Enterobacteriaceae to FEP, for which the susceptible-dose dependent (SDD) interpretive category has replaced the intermediate category, M100-S23 criteria were used to maintain the intermediate category for analysis.

Quality controls were performed on each day of testing using appropriate ATCC control strains, following CLSI and manufacturer guidelines. Results were included in the analysis only when corresponding quality control results were within the acceptable ranges.

### Statistical analysis

Data are expressed as number (n) and percentage (%). χ2 test was used for comparisons. P < 0.05 was considered as the level of statistical significance.

### Ethical aspects

Only isolates were used so the authors never had information that identified patients. As it is not a clinical trial, and no patient identifying data are used or collected. The intra-abdominal and urinary samples were fully anonymized before any of the authors accessed them. According with this kind of study, is not necessary the informed consent and ethical committee, because SMART study only use isolates.

## Results

### Distribution of Gram-negative bacilli

A total of 3,958 Gram-negative bacilli were isolated between 2009 and 2015, of which 57% were *E*. *coli* and 12% were *K*. *pneumoniae* (Table 1). 2,682 of the isolates were collected from IAI and 1,235 from UTI. The number of isolates collected in the National Institutes of Health and in the General Hospitals were similar, respectively 1,975 and 1,983. Overall, after *E*. *coli* and *K*. *pneumoniae*, the most frequent isolates were *Pseudomonas aeruginosa* (*P*. *aeruginosa*), *Acinetobacter baumannii* (*A*. *baumannii*), *Enterobacter cloacae* (*E*. *cloacae*), *P*. *mirabilis* and *K*. *oxytoca*. These species (including the ESBL-producing strains) accounted for 91% of all isolates (n≥70). The trends over time for both the National Institutes of Health and General Hospitals, per IAI and UTI, are available in the Supplementary Information (Tables A and B in S1 Tables).

**Table 1:** Distribution of isolates in the National Institutes of Health and General Hospitals, categorized by intra-abdominal infections, urinary-tract infections and unknown, from SMART study in Mexico between 2009 and 2015.

		National Institutes of Health			General Hospitals		
Pathogen	Overall, n	IAI, n	UTI, n	Unknown, n	IAI, n	UTI, n	Unknown, n
*Escherichia coli*	2274	854	383	19	611	407	0
*Klebsiella pneumoniae*	483	148	67	5	160	100	3
*Pseudomonas aeruginosa*	346	109	56	5	152	24	0
*Acinetobacter baumannii*	206	13	8	0	165	18	2
*Enterobacter cloacae*	148	51	2	1	71	23	0
*Proteus mirabilis*	90	19	23	1	26	21	0
*Klebsiella oxytoca*	70	29	9	0	25	7	0
*Morganella morganii*	63	18	8	2	20	15	0
*Citrobacter freundii*	61	25	11	2	17	6	0
*Serratia marcescens*	36	9	7	0	15	5	0
*Enterobacter aerogenes*	33	19	2	0	7	5	0
*Proteus vulgaris*	23	10	0	0	9	4	0
*Stenotrophomonas maltophilia*	20	7	2	0	9	2	0
*Others*	105	44	7	0	40	13	1
***Total***	**3958**	**1355**	**585**	**35**	**1327**	**650**	**6**

n: number of isolates; IAI: intra-abdominal infections; UTI: urinary-tract infections.

### Prevalence of ESBL-producing organisms

Overall, ESBL-producing organisms accounted for 54% of *E*. *coli* isolates, 39% of *K*. *pneumonia* isolates, 20% of *K*. *oxytoca* isolates, and 2% of *P*. *mirabilis* isolates (Table 2). With the exception of *P*. *mirabilis* that only occurred in UTI, the prevalence of the remaining ESBL-producing organisms was higher for IAI than -UTI. The ESBL-producing *E*. *coli* and *K*. *pneumoniae* isolated from IAI were more prevalent in nosocomial infections than in community-acquired infections (30% *vs*. 25% and 25% *vs*. 15%, respectively). The ESBL-producing organisms isolated from UTI presented the same trend for *E*. *coli*, whereas for *K*. *pneumoniae* the prevalence of ESBL-producing isolates was higher in community-acquired infections than in nosocomial infections (respectively, 22%% *vs*. 13%). The prevalence of the ESBL-producing *K*. *oxytoca* was similar when isolated from IAI (20%) or UTI (19%), and their percentage was always higher in community-acquired infections.

**Table 2:** Prevalence of ESBL-producing organisms by overall, intra-abdominal infection and urinary-tract infection, categorized by type of infection (community-acquired and nosocomial), from SMART study in Mexico between 2009 and 2015.

	Overall						IAI						UTI					
	Total		CA ^a)^		N ^b)^		Total		CA ^a)^		N ^b)^		Total		CA ^a)^		N ^b)^	
ESBL-producing organism	N	%	N	%	n	%	n	%	n	%	n	%	n	%	n	%	n	%
*Escherichia coli*	1225*	54%	525	23%	675	30%	818*	56%	370	25%	437	30%	397*	50%	155	20%	238	30%
*Klebsiella pneumoniae*	186*	39%	84	18%	97	20%	124*	40%	47	15%	76	25%	59*	35%	37	22%	21	13%
*Klebsiella oxytoca*	14	20%	8	11%	6	9%	11	20%	6	11%	5	9%	3	19%	2	13%	1	6%
*Proteus mirabilis*	2	2%	1	1%	1	1%	0	0%	0	0%	0	0%	2	5%	1	2%	1	2%

The prevalence of ESBL-producing organisms were calculated for the total of each organism in the overall intra-abdominal infection (IAI) and urinary-tract infection (UTI), as presented in Table 1;

^a)^ Community-acquired (CA) was defined as an isolate obtained <48h after hospitalization;

^b)^ Nosocomial (N) was defined as an isolate obtained >48h after hospitalization;

*For some isolates herein included the type of infection and the type of source were not specified.

The prevalence of ESBL-producing *E*. *coli* and *K*. *pneumoniae* was slightly variable through the years, respectively ranging from 41%-65% and 30%-47% for IAI, and from 40%-57% and 25%-49% for UTI (S1 Fig). Though ESBL-producing *K*. *pneumoniae* presented slightly lower prevalence when compared to ESBL-producing *E*. *coli*, each species had similar ranges for IAI and UTI. During the study period, 11 out of 54 *K*. *oxytoca* isolates collected from IAI were ESBL-producing bacteria, and their prevalence were irregular over the years (ranging from 0%-38%). Likewise, only 3 out of 16 isolates found in UTI were ESBL-producing *K*. *oxytoca*; two of them were found in 2009 (50%) and one in 2014 (100%). ESBL-producing *P*. *mirabilis* was only reported for two isolates from UTI, one found in 2012 (33%) and the other in 2015 (8%).

### Overall antimicrobial susceptibility

The antimicrobial susceptibility of the 6 most frequent species isolated from IAI and UTI in all study sites between 2009 and 2015 are presented in Table 3. Overall, the classes of antibiotics that showed a preserved bacterial susceptibility were the two carbapenems (ETP and IMP) and the aminoglycoside AMK. There were, however, some exceptions such as the case of the ESBL and non-ESBL-producing *P*. *mirabilis* that were, non- and low-susceptible to IMP, respectively (0% and 35%). Additionally, the susceptibility rates for *P*. *aeruginosa* and *A*. *baumannii* were low for most of the tested antibiotics. In general, FOX was the cephalosporin to which all isolates presented the highest susceptibility rates, except for *E*. *cloacae*. Regarding the other two β-lactams evaluated, all isolates, except *A*. *baumannii*, have shown higher susceptibility to TZP than to SAM. This was especially noted for the ESBL-producing *K*. *oxytoca* and *P*. *aeruginosa* that presented no susceptibility for the latter. The susceptibility rate for both fluoroquinolones (LVX and CIP) were mostly higher than 50%, except for *E*. *coli*, *A*. *baumannii* and some of the ESBL-producing isolates. In general, the non-ESBL-producing isolates presented higher susceptibility to all antibiotics when compared with their corresponding ESBL-producing ones. Moreover, with the exception of FOX, the ESBL-producing isolates showed negligible susceptibility to the remaining cephalosporins.

**Table 3:** Antimicrobial susceptibilities of the most common isolates including the ESBL-producing ones, from intra-abdominal infections and urinary-tract infections, from SMART study in Mexico from 2009 to 2015.

Pathogen	% Susceptibility											
	ETP	IMP	TZP	SAM	FOX	CAZ	CRO	CTX	FEP	LVX	CIP	AMK
*Escherichia coli*	99	99	87	22	79	50	45	45	48	37	36	97
*Escherichia coli*, *ESBL*	99	99	81	6	72	9	1	1	1	11	11	94
*Escherichia coli*, *non ESBL*	100	99	92	37	85	90	90	90	96	62	60	99
*Klebsiella pneumoniae*	97	98	85	47	88	62	56	57	60	80	64	96
*Klebsiella pneumoniae*, *ESBL*	98	100	70	7	87	13	0	1	7	64	27	93
*Klebsiella pneumoniae*, *non ESBL*	97	96	94	77	89	95	92	94	97	93	90	98
*Pseudomonas aeruginosa*	0	62	60	0	0	59	0	0	61	58	59	70
*Acinetobacter baumannii*	0	23	11	25	0	11	6	7	9	9	6	19
*Enterobacter cloacae*	84	98	67	25	5	55	51	52	67	89	85	93
*Proteus mirabilis*	100	34	98	76	98	98	92	96	94	89	72	100
*Proteus mirabilis*, *ESBL*	100	0	100	50	100	50	0	0	50	50	0	100
*Proteus mirabilis*, *non ESBL*	100	35	98	76	98	100	94	98	96	90	74	100
*Klebsiella oxytoca*	100	96	90	57	93	82	75	77	82	74	73	100
*Klebsiella oxytoca*, *ESBL*	100	100	69	0	85	23	0	0	8	23	15	100
*Klebsiella oxytoca*, *non ESBL*	100	95	96	71	94	97	94	97	99	87	87	100

ETP: ertapenem, IMP: imipenem, TZP: Piperacillin-Tazobactam, SAM: Ampicillin-Sulbactam, FOX: cefoxitin, CAZ: ceftazidime, CRO: ceftriaxone, CTX: cefotaxime, FEP: cefepime, LVX: levofloxacin, CIP: ciprofloxacin and AMK: amikacin. These MIC breakpoints have not been defined by the Clinical and Laboratory Standards Institute.

### Antimicrobial susceptibility by IAI and UTI

The susceptibility of these isolates was also analysed by IAI and UTI (Tables 4 and 5), for all study sites, between 2009 and 2015, taking into account the type of infection (community-acquired or nosocomial). Although some differences could be observed, in general, the antimicrobial susceptibility trend for each isolate was similar for IAI and UTI.

**Table 4:** Antimicrobial susceptibilities of the most common isolates including the ESBL-producing ones for the National Institutes of Health and General Hospitals, from intra-abdominal infections, from SMART study in Mexico from 2009 to 2015.

Pathogen		% Susceptibility from intra-abdominal infections											
	Type of infection ^a)^	ETP	IMP	TZP	SAM	FOX	CAZ	CRO	CTX	FEP	LVX	CIP	AMK
*Escherichia coli*	CA	98	99	86	27	78	46	41	41	45	35	35	97
	N	99	99	86	19	78	54	49	49	50	39	38	97
*Escherichia coli*, *ESBL*	CA	97	98	80	8	73	8	0	0	1	13	13	94
	N	99	99	79	3	69	11	1	1	1	9	8	93
*Escherichia coli*, *non ESBL*	CA	100	100	91	44	81	86	85	84	93	61	61	99
	N	100	99	94	34	89	95	95	95	98	67	64	99
*Klebsiella pneumoniae*	CA	99	99	85	52	88	64	63	64	64	84	66	100
	N	96	98	82	46	91	60	52	52	57	74	59	94
*Klebsiella pneumoniae*, *ESBL*	CA	98	100	65	5	83	2	1	1	1	70	20	99
	N	97	100	68	7	91	19	0	2	10	52	22	92
*Klebsiella pneumoniae*, *non ESBL*	CA	99	99	97	83	94	99	98	99	99	97	97	100
	N	98	97	94	80	91	95	94	95	98	94	90	98
*Pseudomonas aeruginosa*	CA	0	61	59	0	0	62	0	0	62	52	52	73
	N	0	65	63	0	0	58	0	0	62	66	67	71
*Acinetobacter baumannii*	CA	0	32	21	43	0	21	7	12	18	11	2	29
	N	0	22	8	20	0	8	8	7	8	11	10	17
*Enterobacter cloacae*	CA	80	99	68	19	0	48	48	51	61	93	84	96
	N	86	96	66	33	8	57	51	50	69	93	91	97
*Proteus mirabilis*	CA	100	31	100	79	100	100	96	96	100	100	92	100
	N	100	39	95	70	95	100	100	100	90	84	67	100
*Proteus mirabilis*, *ESBL*	CA	-	-	-	-	-	-	-	-	-	-	-	-
	N	-	-	-	-	-	-	-	-	-	-	-	-
*Proteus mirabilis*, *non ESBL*	CA	100	31	100	79	100	100	96	96	100	100	92	100
	N	100	39	95	70	95	100	100	100	90	84	67	100
*Klebsiella oxytoca*	CA	100	100	97	48	95	78	70	70	78	65	65	100
	N	100	95	86	61	93	83	73	78	83	80	78	100
*Klebsiella oxytoca*, *ESBL*	CA	100	100	83	0	83	17	0	0	17	17	17	100
	N	100	100	60	0	80	40	0	0	0	40	20	100
*Klebsiella oxytoca*, *non ESBL*	CA	100	100	98	68	95	98	98	98	98	82	82	100
	N	100	94	92	74	94	94	89	94	100	89	89	100

ETP: ertapenem, IMP: imipenem, TZP: Piperacillin-Tazobactam, SAM: Ampicillin-Sulbactam, FOX: cefoxitin, CAZ: ceftazidime, CRO: ceftriaxone, CTX: cefotaxime, FEP: cefepime, LVX: levofloxacin, CIP: ciprofloxacin and AMK: amikacin.

^a)^ The infections were categorized as community-acquired (CA) and nosocomial (N) defined, respectively, as isolates obtained in <48 hours or >48h after hospitalization; These MIC breakpoints have not been defined by the Clinical and Laboratory Standards Institute.

**Table 5:** Antimicrobial susceptibilities of the most common bacteria including the ESBL-producing ones for the National Institutes of Health and General Hospitals, from urinary-tract infections, from SMART study in Mexico from 2010 to 2015.

Pathogen		% Susceptibility from urinary-tract infections											
	Type of infection ^a)^	ETP	IMP	TZP	SAM	FOX	CAZ	CRO	CTX	FEP	LVX	CIP	AMK
*Escherichia coli*	CA	100	100	83	18	81	48	45	45	47	34	31	98
	N	100	100	88	24	82	50	44	44	49	36	35	96
*Escherichia coli*, *ESBL*	CA	100	100	80	11	78	4	0	0	0	20	18	94
	N	100	100	84	7	70	11	2	0	4	10	8	94
*Escherichia coli*, *non ESBL*	CA	99	99	94	31	85	94	94	94	98	49	44	99
	N	99	99	84	39	86	87	86	86	95	62	61	100
*Klebsiella pneumoniae*	CA	95	95	82	43	84	53	50	50	52	76	57	94
	N	98	98	92	50	84	73	59	64	69	89	78	98
*Klebsiella pneumoniae*, *ESBL*	CA	100	100	72	3	89	9	0	0	3	66	24	88
	N	100	100	80	7	80	20	0	0	14	83	50	100
*Klebsiella pneumoniae*, *non ESBL*	CA	89	89	86	71	80	88	87	87	89	82	82	94
	N	98	98	96	73	85	97	84	90	98	91	88	98
*Pseudomonas aeruginosa*	CA	0	44	44	0	0	45	0	0	49	44	45	56
	N	0	74	80	0	0	74	0	0	75	59	68	77
*Acinetobacter baumannii*	CA	0	40	20	40	0	20	0	0	0	0	0	20
	N	0	6	0	2	0	0	0	0	0	0	0	6
*Enterobacter cloacae*	CA	100	100	100	0	0	100	100	100	100	100	100	100
	N	84	100	63	14	9	56	50	56	69	69	66	78
*Proteus mirabilis*	CA	100	47	100	94	100	100	88	97	100	91	66	100
	N	100	19	98	73	98	90	74	85	90	81	60	100
*Proteus mirabilis*, *ESBL*	CA	100	0	100	0	100	100	0	0	100	100	0	100
	N	100	0	100	100	100	0	0	0	0	0	0	100
*Proteus mirabilis*, *non ESBL*	CA	100	50	100	95	100	100	91	100	100	91	69	100
	N	100	21	98	70	98	98	81	93	98	89	66	100
*Klebsiella oxytoca*	CA	100	100	100	67	83	89	89	89	89	72	72	100
	N	100	75	75	50	100	75	75	75	75	75	75	100
*Klebsiella oxytoca*, *ESBL*	CA	100	100	100	0	100	0	0	0	0	0	0	100
	N	100	100	0	0	100	0	0	0	0	0	0	100
*Klebsiella oxytoca*, *non ESBL*	CA	100	100	100	67	83	100	100	100	100	83	83	100
	N	100	67	100	67	100	100	100	100	100	100	100	100

ETP: ertapenem, IMP: imipenem, TZP: Piperacillin-Tazobactam, SAM: Ampicillin-Sulbactam, FOX: cefoxitin, CAZ: ceftazidime, CRO: ceftriaxone, CTX: cefotaxime, FEP: cefepime, LVX: levofloxacin, CIP: ciprofloxacin and AMK: amikacin.

^a)^ The infections were categorized as community-acquired (CA) and nosocomial (N) defined, respectively, as isolates obtained in <48 hours or >48h after hospitalization; These MIC breakpoints have not been defined by the Clinical and Laboratory Standards Institute.

ESBL-producing *K*. *oxytoca* showed lower susceptibility towards the tested fluoroquinolones and cephalosporins when isolated from UTI than from IAI, except for FOX that reported a susceptibility of 100% for UTI and only around 80% for IAI. In addition, ESBL-producing *K*. *oxytoca* isolated from nosocomial UTIs were not susceptible to TZP, but presented a 60% susceptibility when isolated from nosocomial IAI. For these classes of antibiotics, *A*. *baumannii* presented a similar susceptibility profile as that of *K*. *oxytoca* in UTI and IAI. However, it presented a lower susceptibility for the remaining antibiotics tested regardless of the isolates source. Additionally, *E*. *cloacae* showed, in general, slightly higher antibiotic susceptibility in UTI than in IAI.

Regarding the type of infection, slight differences can also be observed. For UTI, all *K*. *pneumoniae* isolates presented higher susceptibility rates for nosocomial than for community-acquired infections. For IAI, isolates from this species showed, for most cases, an opposite behavior. In contrast, *E*. *cloacae* and *P*. *mirabilis* showed mostly lower susceptibility values for nosocomial UTIs, and a contrary behavior for IAIs. In addition, *P*. *aeruginosa* presented higher susceptibility rates for community-acquired IAIs when compared to UTIs, and an opposite trend for the susceptibility rates for nosocomial infections.

### Antimicrobial susceptibility by National Institutes of Health and General Hospitals

Similar antimicrobial susceptibility trends were found for these isolates when comparing their profiles by type of healthcare institution. The major differences were observed for ESBL-producing *K*. *oxytoca* and for *P*. *aeruginosa* isolates. Data on the susceptibility of these isolates by the National Institutes of Health and General Hospitals, isolated from IAI and UTI, between 2009 and 2015, and taking into account the type of infection can be found in Supplementary Information Tables C and D in S1 Tables.

Overall, ESBL-producing *K*. *oxytoca* isolates obtained from community-acquired infections were more susceptible in the National Institutes than in the General Hospitals. When obtained in the General Hospitals, these isolates only presented susceptibility for the tested carbapenems (ETP and IMP) and for the aminoglycoside AMK, and the ones isolated in the National Institutes were also susceptible to TZP (100%), FOX (100%), and -in a lower extent to CAZ, FEP, LVX and CIP (all with 17% susceptibility). *P*. *aeruginosa* showed the same susceptibility trend for all antibiotics, with slightly higher values for isolates obtained in the General Hospitals, when compared with the ones obtained in the National Institutes. In addition, the two ESBL-producing *P*. *mirabilis* isolates from UTI, reported in this study, were recorded in the National Institutes.

### Antimicrobial susceptibility trends for *E*. *coli* and *K*. *pneumoniae*

As the antimicrobial susceptibility profiles of ESBL and non-ESBL producing *E*. *coli* and *K*. *pneumoniae* have presented similar trends for IAI and UTI, and both types of healthcare institutions, their overall susceptibility over the years was presented for all the tested antibiotics, in (S2 and S3 Figs). The ESBL-producing *E*. *coli* and *K*. *pneumoniae* were more susceptible to the two carbapenems (ETP and IMP) and the aminoglycoside AMK. They were also susceptible to FOX, however ESBL-producing *E*. *coli*’s susceptibility rates have been decreasing since 2012. These isolates are still susceptible to TZP within the range of 76%-85%. The susceptibility of the ESBL-producing *K*. *pneumoniae* to TZP and LVX was widely variable throughout the years, ranging from 39%-88% and 29%-82%, respectively. Non-ESBL-producing isolates have shown high susceptibility to most of the antibiotics, except for SAM with values ranging from 18%-49% and 54%-8% for E.coli and K. pneumoniae, respectively. FOX was the cephalosporin that presented lower susceptibility rates for these non-ESBL-producing isolates. Contrary to the non-ESBL-producing *K*. *pneumoniae* that is still susceptible to the tested fluoroquinolones (≥87% for LVX and CIP), the non-ESBL-producing *E*. *coli* showed low susceptibility with a decreasing tendency over the last years.

## Discussion

Overall, the results from these 7 years of surveillance period (2009 to 2015) in Mexico reinforced the trends of what has been reported in Latin America so far [12–14]. Both *E*. *coli* and *K*. *pneumoniae* revealed to be the most frequently reported organisms, as well as the ones with the highest prevalence of ESBL-producing isolates. Since their identification in Germany in the early 1980s [18], the increase in ESBL-producing organisms has been a worldwide concern, as their reduced antimicrobial susceptibility hampers treatment options. Several surveillance studies have shown that the raise in ESBL-producing organisms is particular high in Latin American and Asian countries, where their prevalence reached values over 50%, within those isolated from IAI [14, 19, 20].

The ESBL rates herein reported for Mexico, 36% for IAI (953/2,682) and 37% for UTI (461/1,235), were slightly higher than the ones reported for Latin America from 2002 to 2011 in the SMART study [12]. In this analysis, ESBL rates for IAI in Latin America were seen to be steadily increasing over time, while for UTI the increase was not significant. For both infections, ESBL rates were lower than 30% [12]. Previous results from SMART assessments have shown that the prevalence of ESBL-producing *E*. *coli* and *K*. *pneumoniae* isolated from IAI in Latin America were increasing over time, respectively 10% *vs*. 14% in 2003 [21], 10% *vs*. 18% in 2004 [22], and 26% *vs*. 35% in 2008 [14]. Overall, our results show that for IAI the rates of ESBL producers were higher (56% for *E*. *coli* and 40% for *K*. *pneumoniae*) when compared to the ones reported in Latin America. Moreover, conversely to the trends observed in the region, in Mexico the rate of ESBL-producing isolates was higher for *E*. *coli* than for *K*. *pneumoniae*. Nevertheless, these results are not completely surprising, given that Mexico has stood out has having one of the highest ESBL rates in Latin America. Results from the SENTRY surveillance program revealed that the maximum rate of ESBL-producing isolates found in Latin America was reported in Mexico for *K*. *pneumoniae* (52%) [23]. A recent update of this study has shown that, from 2008 to 2010, the rates of ESBL-producing isolates were 48.4% among *E*.*coli* and 33.3% among *Klebsiella spp*. in Mexico [24]. The results found here are in line with this update of the SENTRY study, further enhancing the growing trend of ESBL-producing organisms.

The prevalence of ESBL-producing organisms was higher in nosocomial infections than in community-acquired infections (21% *vs*. 27%). The results of both infections are similar to those previously reported for Latin America (respectively 31% and 25%) [14], yet the ESBL rate obtained in nosocomial infections was much lower than the one reported in the Asia/Pacific region (55%) [20]. Nevertheless, it is worth mentioning that the SMART classification as community-acquired infection and nosocomial (respectively, isolates obtained within <48h and >48h after hospitalization), might be misleading due to the uncertainty of the time of sampling of the isolates and/or possible prior hospitalizations [13].

In this study, the ESBL-producing organisms were highly resistant to fluoroquinolones, to third and fourth generation cephalosporins, and also to other β-lactams (SAM and TZP in a lower extent). These results are in line with previous worldwide reports of antimicrobial resistance against these classes of antibiotics [16, 25–27], and lead to a lower use of fluoroquinolones as UTI empiric treatment [15, 28]. One exception was denoted for ESBL-producing *P*. *mirabilis* isolated from community-acquired UTIs, which still reported 100% susceptibility for CAZ, FEP and LVX. However, care should be taken when analyzing these results, as ESBL-producing *P*. *mirabilis* was only reported for two isolates in this study. As a consequence of the several reports from our country, recommendations for empiric antibiotic use in urinary tract infections have been issued recently [29].

Most of the Gram-negative bacilli, isolated from IAI and UTI, were susceptible to the carbapenems tested, thus preserving the consistency of the results obtained since the beginning of the SMART study [12, 13]. AMK was the following antibiotic more active against most of the isolates. Interesting, the susceptibility of ESBL-producing K. pneumoniae seams to increase over the last years. A similar trend was observed for this aminoglycoside from 2005 to 2010 in North America [12, 13]. These results are particularly important for ESBL-producing organisms, because despite their increasing prevalence, most isolates can still receive appropriate treatment. However, there were some exceptions, for which treatment options are becoming increasingly scarce. *P*. *aeruginosa* and *A*. *baumannii* were the two isolates that presented, in general, lower susceptibility rates to the tested antibiotics. Nevertheless, *A*. *baumannii* presented much lower susceptibility rates than *P*. *aeruginosa*, with maximum rates averaging 43% for IAI, 40% for UTI, 49% for the National Institutes and 35% for General Hospitals. These alarming results were already predictable, since *A*. *baumannii* was already being reported in Mexico, and it is recognized as one of the most difficult antimicrobial-resistant Gram-negative bacilli to control and treat [30,31].

## Conclusions

Despite the intrinsic limitations of a worldwide surveillance study, some important issues should be pointed out. The SMART study comprise over 50 countries worldwide, and about 180 sites [12]. In the particular case of Mexico, 4 sites are considered in this analysis; 2 hospitals in the north and center-west, and 2 National Institutes in Mexico City. Therefore, care should be taken when extrapolating these results towards other Mexican regions, as the antimicrobial susceptibility is widely variable even within different hospital admission services. Nevertheless, the continuous surveillance of antimicrobial trends is vital to guide physicians in the effective empiric antimicrobial treatment for UTI and IAI, as has led to the creation of the national action plan to prevent antimicrobial resistance in accordance to the WHO guide. In this particular plan, special emphasis has been placed on the laboratory component of the global plan, as well as the inclusion of this evidence in the strategic planning of antimicrobial stewardship programs [32]. (Alfredo Ponce-de-Leon / José Sifuentes Osornio, personal communication).

The results herein obtain are in line with the global trends, though further enhancing the increased rates observed in Mexico, when compared with the global Latin America reality. One of the major global concerns regarding antimicrobial resistance is the incessantly rise of ESBL-producing organisms, especially the ones highly resistant. Moreover, there is a growing need to develop effective treatment options, both new drug discovery and new combinations of already existing antimicrobials, as well as to ensure the prevention of antimicrobial resistant infections.

## Supporting information

S1 Fig Prevalence of ESBL in E. coli, K. Pneumonia, K. oxytoca and P. mirabilis from a) intra-abdominal infections and b) urinary-tract infections, from SMART study in Mexico between 2009 and 2015. (TIFF)

S2 Fig Antimicrobial susceptibilities of a) ESBL-producing E. coli and b) non ESBL-producing E. coli, from intra-abdominal infections and urinary-tract infections, from SMART study in Mexico from 2009 to 2015. (TIFF)

S3 Fig Antimicrobial susceptibilities of a) ESBL-producing K. pneumoniae and b) non ESBL-producing K. pneumoniae, from SMART study in Mexico from 2009 to 2015. Susceptibilities were based on in vitro minimum inhibitory concentration data tested for the following antimicrobials classes: carbapenems (ETP: ertapenem, IMP: imipenem), other β-lactam antibiotics (TZP: Piperacillin-Sulbactam, SAM: Ampicillin-Sulbactam), cephalosporins (FOX: cefoxitin, CAZ: ceftazidime, CRO: ceftriaxone, CTX: cefotaxime, FEP: cefepime), Fluoroquinolones (LVX: levofloxacin, CIP: ciprofloxacin) and aminoglycosides (AMK: amikacin).
(TIFF)

S1 Tables Distribution of isolates from intra-abdominal infections, urinary-tract infections by National Institutes of Health and General Hospitals per year, from SMART study in Mexico between 2009 and 2015 (A and B respectively). Antimicrobial susceptibilities of the most common isolates including the ESBL-producing ones for the National Institutes of Health, and the General Hospitals from intra-abdominal infections and urinary-tract infections, from SMART study in Mexico from 2009 to 2015 (C and D respectively).
(DOCX)

S1 Dataset Antibiogram 2009 a 2015 IAI and UTI. (ZIP)

## References

[1] Cohen AE, Lautenbach E, Morales KH, Linkin DR. 2006 Fluoroquinolone-resistant Escherichia coli in the long-term care setting. Am J Med 119:958–63. doi: 10.1016/j.amjmed.2006.05.030 17071164 10.1016/j.amjmed.2006.05.030

[2] Paterson DL, Bonomo RA. 2005 Extended-spectrum beta-lactamases: a clinical update. Clin Microbiol Rev 18:657–86. doi: 10.1128/CMR.18.4.657-686.2005 16223952 10.1128/CMR.18.4.657-686.2005 PMC1265908

[3] Rawat D, Nair D. 2010 Extended-spectrum β-lactamases in Gram Negative Bacteria. Journal of Global Infectious Diseases 2:263–274. doi: 10.4103/0974-777X.68531 20927289 10.4103/0974-777X.68531 PMC2946684

[4] Gupta N, Limbago BM, Patel JB, Kallen AJ. 2011 Carbapenem-resistant Enterobacteriaceae: epidemiology and prevention. Clin Infect Dis 53:60–7. doi: 10.1093/cid/cir202 21653305 10.1093/cid/cir202

[5] Perez F, Van Duin D. 2013 Carbapenem-resistant Enterobacteriaceae: A menace to our most vulnerable patients. Cleveland Clinic journal of medicine 80:225–233. doi: 10.3949/ccjm.80a.12182 23547093 10.3949/ccjm.80a.12182 PMC3960994

[6] Torres-Gonzalez P, Cervera-Hernandez ME, Niembro-Ortega MD, Leal-Vega F, Cruz-Hervert LP, Garcia-Garcia L, et al 2015 Factors Associated to Prevalence and Incidence of Carbapenem-Resistant Enterobacteriaceae Fecal Carriage: A Cohort Study in a Mexican Tertiary Care Hospital. PLoS One 10:e0139883 doi: 10.1371/journal.pone.0139883 26431402 10.1371/journal.pone.0139883 PMC4592225

[7] Salles MJC, Zurita J, MejÍA C, Villegas MV. 2013 Resistant Gram-negative infections in the outpatient setting in Latin America. Epidemiology and Infection 141:2459–2472. doi: 10.1017/S095026881300191X 23924513 10.1017/S095026881300191X PMC3821403

[8] Pitout JD, Laupland KB. 2008 Extended-spectrum beta-lactamase-producing Enterobacteriaceae: an emerging public-health concern. Lancet Infect Dis 8:159–66. doi: 10.1016/S1473-3099(08)70041-0 18291338 10.1016/S1473-3099(08)70041-0

[9] Kang CI, Song JH, Chung DR, Peck KR, Ko KS, Yeom JS, et al 2010 Risk factors and treatment outcomes of community-onset bacteraemia caused by extended-spectrum beta-lactamase-producing Escherichia coli. Int J Antimicrob Agents 36:284–7. doi: 10.1016/j.ijantimicag.2010.05.009 20580534 10.1016/j.ijantimicag.2010.05.009

[10] Rodriguez-Bano J, Picon E, Gijon P, Hernandez JR, Ruiz M, Pena C, et al 2010 Community-onset bacteremia due to extended-spectrum beta-lactamase-producing Escherichia coli: risk factors and prognosis. Clin Infect Dis 50:40–8. doi: 10.1086/649537 19995215 10.1086/649537

[11] Hawser S. 2012 Surveillance programmes and antibiotic resistance: worldwide and regional monitoring of antibiotic resistance trends. Handb Exp Pharmacol doi: 10.1007/978-3-642-28951-4_3:31–43. 23090594 10.1007/978-3-642-28951-4_3

[12] Morrissey I, Hackel M, Badal R, Bouchillon S, Hawser S, Biedenbach D. 2013 A Review of Ten Years of the Study for Monitoring Antimicrobial Resistance Trends (SMART) from 2002 to 2011. Pharmaceuticals 6:1335–1346. doi: 10.3390/ph6111335 24287460 10.3390/ph6111335 PMC3854014

[13] Baquero F, Hsueh PR, Paterson DL, Rossi F, Bochicchio GV, Gallagher G, et al 2009 In vitro susceptibilities of aerobic and facultatively anaerobic gram-negative bacilli isolated from patients with intra-abdominal infections worldwide: 2005 results from Study for Monitoring Antimicrobial Resistance Trends (SMART). Surg Infect (Larchmt) 10:99–104. 18831681 10.1089/sur.2008.0020

[14] Villegas MV, Blanco MG, Sifuentes-Osornio J, Rossi F. 2011 Increasing prevalence of extended-spectrum-beta-lactamase among Gram-negative bacilli in Latin America—2008 update from the Study for Monitoring Antimicrobial Resistance Trends (SMART). Braz J Infect Dis 15:34–9. 21412587

[15] Bouchillon SK, Badal RE, Hoban DJ, Hawser SP. 2013 Antimicrobial susceptibility of inpatient urinary tract isolates of gram-negative bacilli in the United States: results from the study for monitoring antimicrobial resistance trends (SMART) program: 2009–2011. Clin Ther 35:872–7. doi: 10.1016/j.clinthera.2013.03.022 23623624 10.1016/j.clinthera.2013.03.022

[16] Jean SS, Coombs G, Ling T, Balaji V, Rodrigues C, Mikamo H, et al 2016 Epidemiology and antimicrobial susceptibility profiles of pathogens causing urinary tract infections in the Asia-Pacific region: Results from the Study for Monitoring Antimicrobial Resistance Trends (SMART), 2010–2013. Int J Antimicrob Agents 47:328–34. doi: 10.1016/j.ijantimicag.2016.01.008 27005459 10.1016/j.ijantimicag.2016.01.008

[17] Kouchak F, Askarian M. 2012 Nosocomial Infections: The Definition Criteria. Iranian Journal of Medical Sciences 37:72–73. 23115435 PMC3470069

[18] Knothe H, Shah P, Krcmery V, Antal M, Mitsuhashi S. 1983 Transferable resistance to cefotaxime, cefoxitin, cefamandole and cefuroxime in clinical isolates of Klebsiella pneumoniae and Serratia marcescens. Infection 11:315–7. 6321357 10.1007/BF01641355

[19] Hsueh PR, Badal RE, Hawser SP, Hoban DJ, Bouchillon SK, Ni Y, et al 2010 Epidemiology and antimicrobial susceptibility profiles of aerobic and facultative Gram-negative bacilli isolated from patients with intra-abdominal infections in the Asia-Pacific region: 2008 results from SMART (Study for Monitoring Antimicrobial Resistance Trends). Int J Antimicrob Agents 36:408–14. doi: 10.1016/j.ijantimicag.2010.07.002 20728316 10.1016/j.ijantimicag.2010.07.002

[20] Hawser SP, Bouchillon SK, Hoban DJ, Badal RE, Hsueh PR, Paterson DL. 2009 Emergence of high levels of extended-spectrum-beta-lactamase-producing gram-negative bacilli in the Asia-Pacific region: data from the Study for Monitoring Antimicrobial Resistance Trends (SMART) program, 2007. Antimicrob Agents Chemother 53:3280–4. doi: 10.1128/AAC.00426-09 19506060 10.1128/AAC.00426-09 PMC2715591

[21] Paterson DL, Rossi F, Baquero F, Hsueh PR, Woods GL, Satishchandran V, et al 2005 In vitro susceptibilities of aerobic and facultative Gram-negative bacilli isolated from patients with intra-abdominal infections worldwide: the 2003 Study for Monitoring Antimicrobial Resistance Trends (SMART). J Antimicrob Chemother 55:965–73. doi: 10.1093/jac/dki117 15849262 10.1093/jac/dki117

[22] Rossi F, Baquero F, Hsueh PR, Paterson DL, Bochicchio GV, Snyder TA, et al 2006 In vitro susceptibilities of aerobic and facultatively anaerobic Gram-negative bacilli isolated from patients with intra-abdominal infections worldwide: 2004 results from SMART (Study for Monitoring Antimicrobial Resistance Trends). J Antimicrob Chemother 58:205–10. doi: 10.1093/jac/dkl199 16717055 10.1093/jac/dkl199

[23] Sader HS, Gales AC, Granacher TD, Pfaller MA, Jones RN, Group SS. 2000 Prevalence of antimicrobial resistance among respiratory tract isolates in Latin America: results from SENTRY antimicrobial surveillance program (1997–98). Braz J Infect Dis 4:245–54. 11063556

[24] Gales AC, Castanheira M, Jones RN, Sader HS. Antimicrobial resistance among Gram-negative bacilli isolated from Latin America: results from SENTRY Antimicrobial Surveillance Program (Latin America, 2008–2010). Diagnostic Microbiology and Infectious Disease 73:354–360. doi: 10.1016/j.diagmicrobio.2012.04.007 22656912 10.1016/j.diagmicrobio.2012.04.007

[25] Lautenbach E, Strom BL, Bilker WB, Patel JB, Edelstein PH, Fishman NO. 2001 Epidemiological investigation of fluoroquinolone resistance in infections due to extended-spectrum beta-lactamase-producing Escherichia coli and Klebsiella pneumoniae. Clin Infect Dis 33:1288–94. doi: 10.1086/322667 11565067 10.1086/322667

[26] Hawser SP, Bouchillon SK, Hoban DJ, Badal RE. 2010 Epidemiologic trends, occurrence of extended-spectrum beta-lactamase production, and performance of ertapenem and comparators in patients with intra-abdominal infections: analysis of global trend data from 2002–2007 from the SMART study. Surg Infect (Larchmt) 11:371–8. 20695829 10.1089/sur.2009.057

[27] Shaikh S, Fatima J, Shakil S, Rizvi SMD, Kamal MA. 2015 Antibiotic resistance and extended spectrum beta-lactamases: Types, epidemiology and treatment. Saudi Journal of Biological Sciences 22:90–101. doi: 10.1016/j.sjbs.2014.08.002 25561890 10.1016/j.sjbs.2014.08.002 PMC4281622

[28] Hoban DJ, Lascols C, Nicolle LE, Badal R, Bouchillon S, Hackel M, et al 2012 Antimicrobial susceptibility of Enterobacteriaceae, including molecular characterization of extended-spectrum beta-lactamase-producing species, in urinary tract isolates from hospitalized patients in North America and Europe: results from the SMART study 2009–2010. Diagn Microbiol Infect Dis 74:62–7. doi: 10.1016/j.diagmicrobio.2012.05.024 22763019 10.1016/j.diagmicrobio.2012.05.024

[29] Sotomayor de Zavaleta M, Ponce de Leon Garduño A, Guzmán Esquivel J, Rosas Nava E, Rodríguez Covarrubias FT, González Ramírez A, et al Recomendaciones de expertos mexicanos en el tratamiento de las infecciones del tracto urinario en pacientes adultos, embarazadas y niños. Rev Mex Urol 2015;75(2):1–47

[30] Alcantar-Curiel MD, Garcia-Torres LF, Gonzalez-Chavez MI, Morfin-Otero R, Gayosso-Vazquez C, Jarillo-Quijada MD, et al 2014 Molecular mechanisms associated with nosocomial carbapenem-resistant Acinetobacter baumannii in Mexico. Arch Med Res 45:553–60. doi: 10.1016/j.arcmed.2014.10.006 25450581 10.1016/j.arcmed.2014.10.006

[31] Maragakis LL, Perl TM. 2008 Acinetobacter baumannii: epidemiology, antimicrobial resistance, and treatment options. Clin Infect Dis 46:1254–63. doi: 10.1086/529198 18444865 10.1086/529198

[32] Federación, D.P. (02 de 04 de 2014). Reglamento de la Ley General de Salud en Materia de investigación para la salud. Accesed on August 30 of 2017. Secretaría de Salud: http://www.cofepris.gob.mx/MJ/Documents/Reglamentos/infestigsalud060187.pdf

