# Supplementary figures and images for: Antimicrobial susceptibility of gram-negative bacilli isolated from intra-abdominal and urinary-tract infections in Mexico from 2009 to 2015: Results from the Study for Monitoring Antimicrobial Resistance Trends (SMART)
Source: PLoS One. 2018 Jun 21;13(6):e0198621. doi: 10.1371/journal.pone.0198621 (PMC6013120; doi:10.1371/journal.pone.0198621)

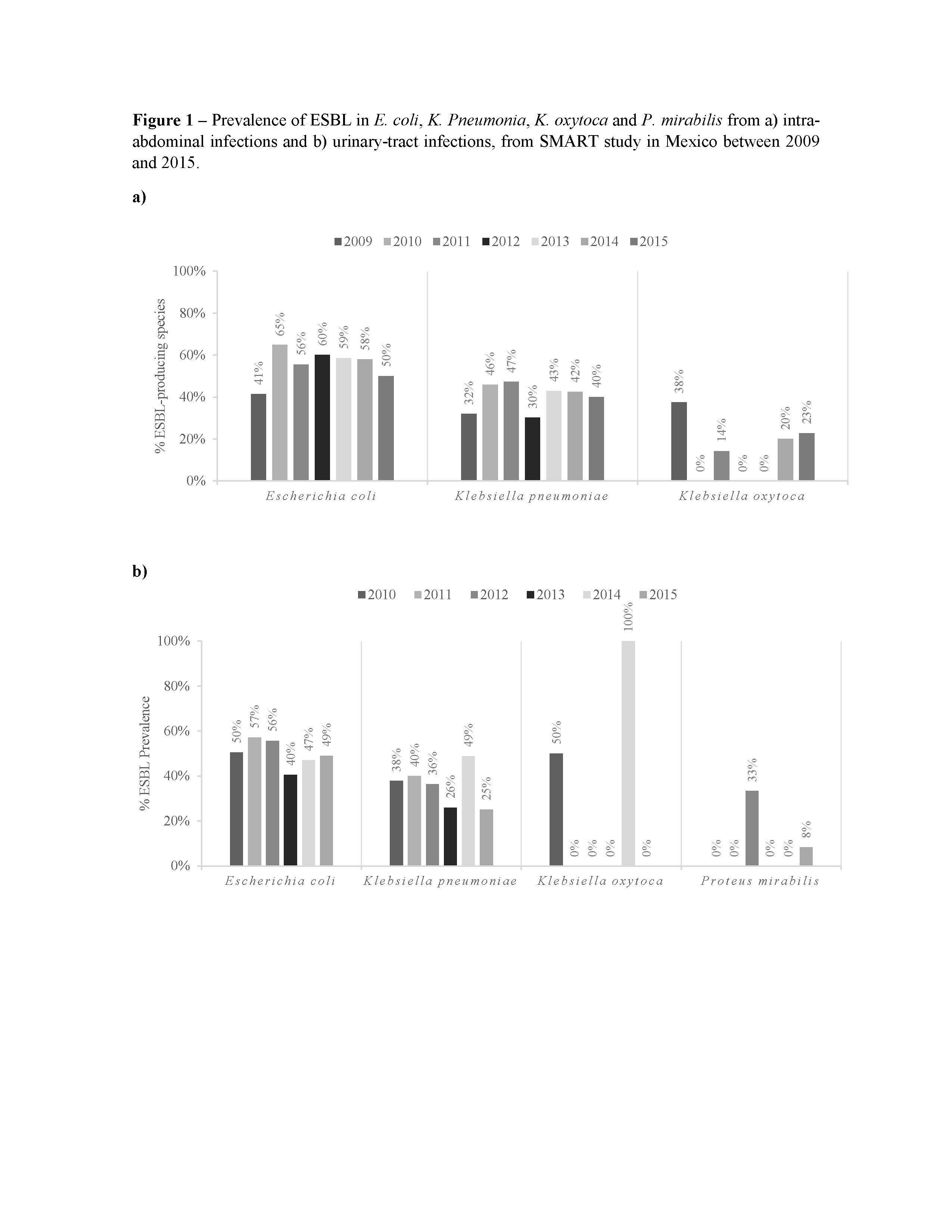

Supplement: S1 Fig — (TIFF) [file pone.0198621.s001.tiff]

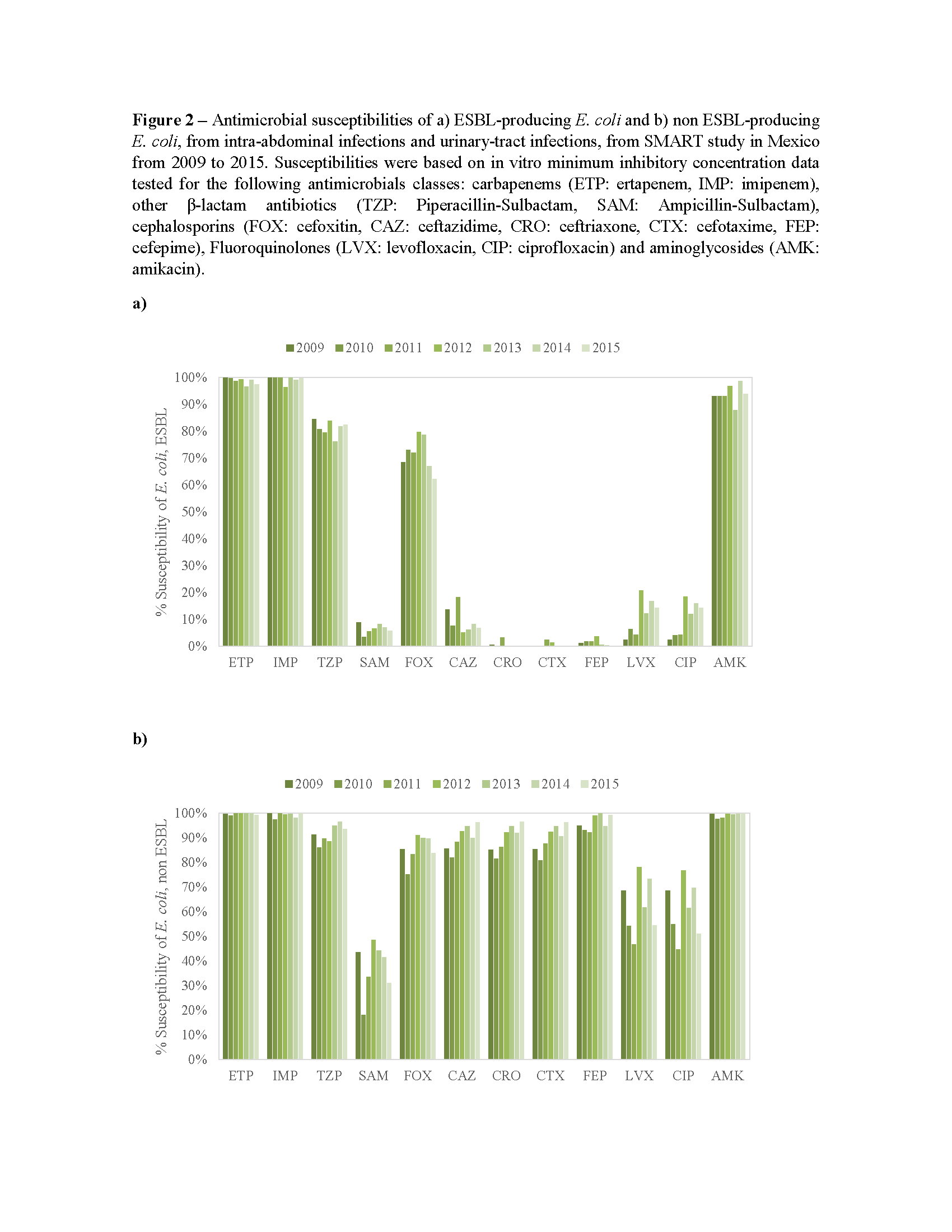

Supplement: S2 Fig — (TIFF) [file pone.0198621.s002.tiff]

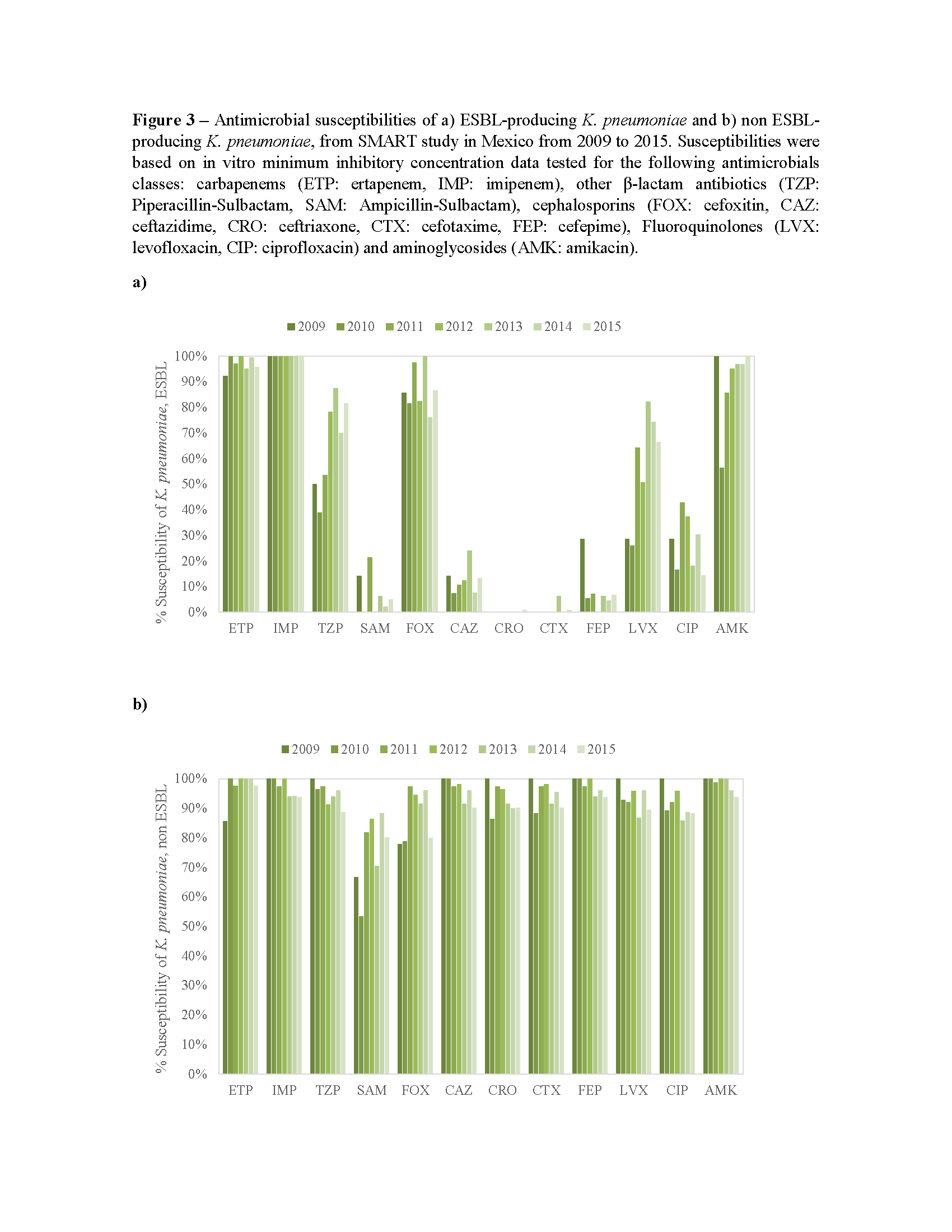

Supplement: S3 Fig — Susceptibilities were based on in vitro minimum inhibitory concentration data tested for the following antimicrobials classes: carbapenems (ETP: ertapenem, IMP: imipenem), other β-lactam antibiotics (TZP: Piperacillin-Sulbactam, SAM: Ampicillin-Sulbactam), cephalosporins (FOX: cefoxitin, CAZ: ceftazidime, CRO: ceftriaxone, CTX: cefotaxime, FEP: cefepime), Fluoroquinolones (LVX: levofloxacin, CIP: ciprofloxacin) and aminoglycosides (AMK: amikacin). (TIFF) [file pone.0198621.s003.tiff]

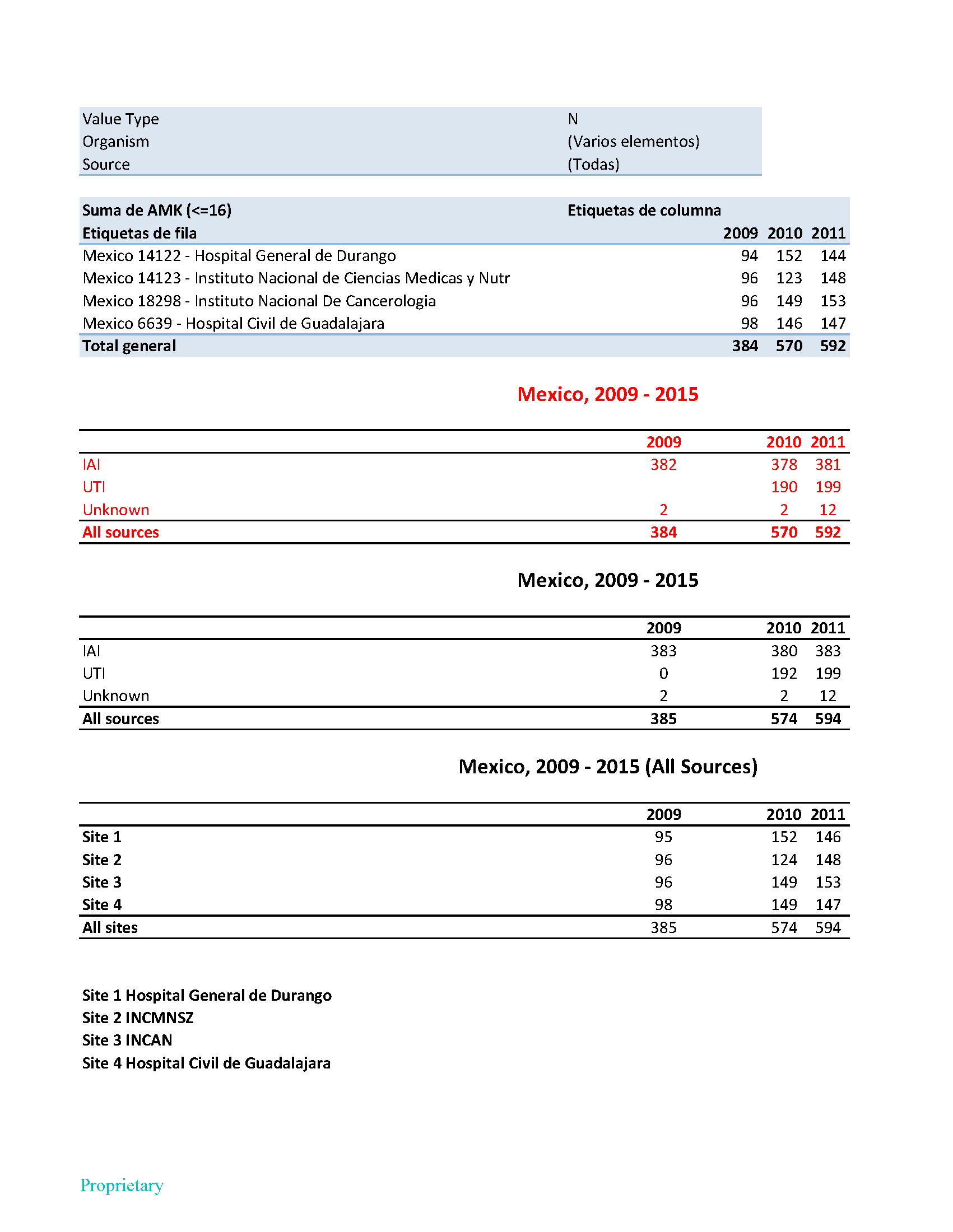

Supplement: S1 Dataset — (ZIP) [file pone.0198621.s005.zip › S1 Data set_Page_1.tiff]

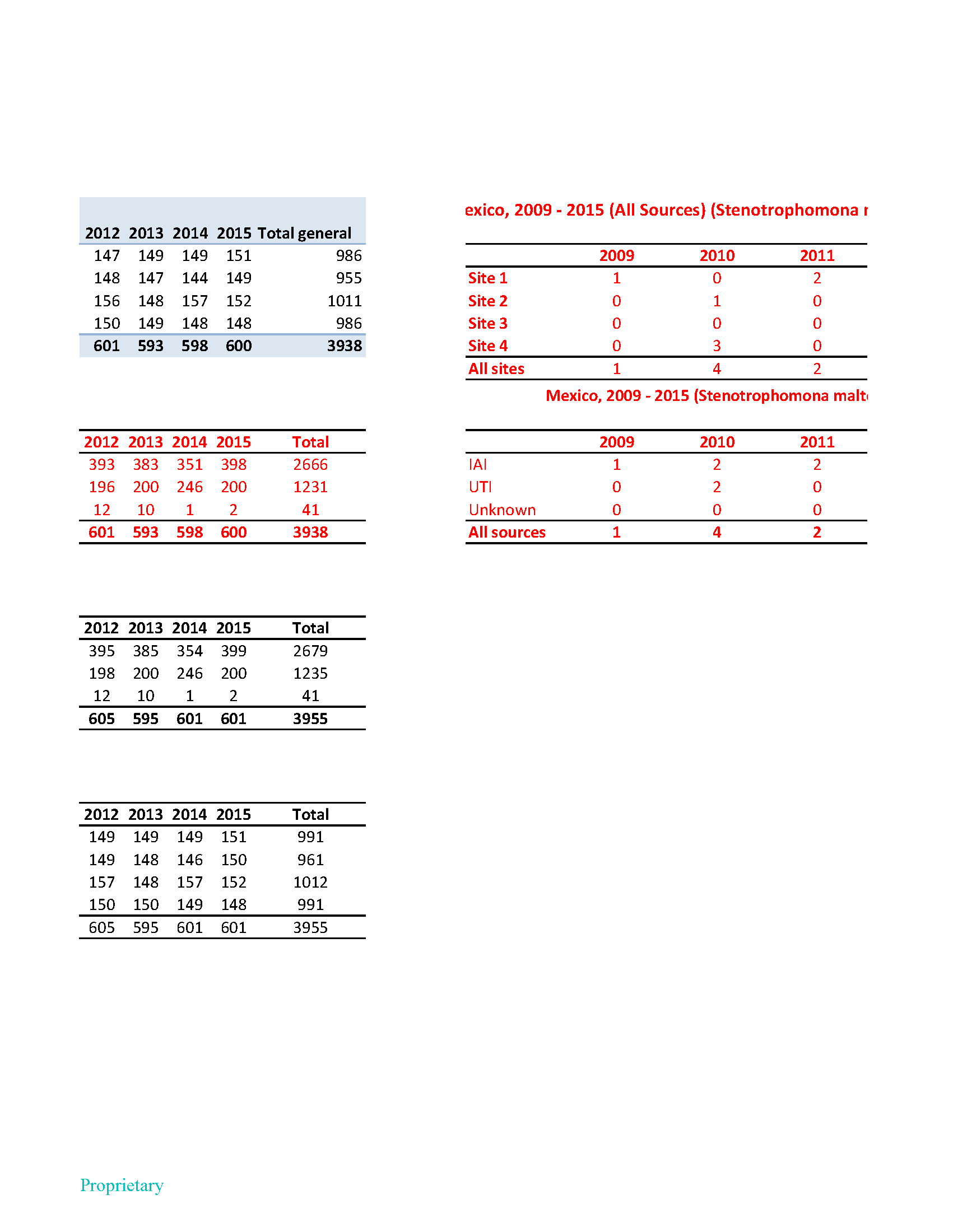

Supplement: S1 Dataset — (ZIP) [file pone.0198621.s005.zip › S1 Data set_Page_2.tiff]

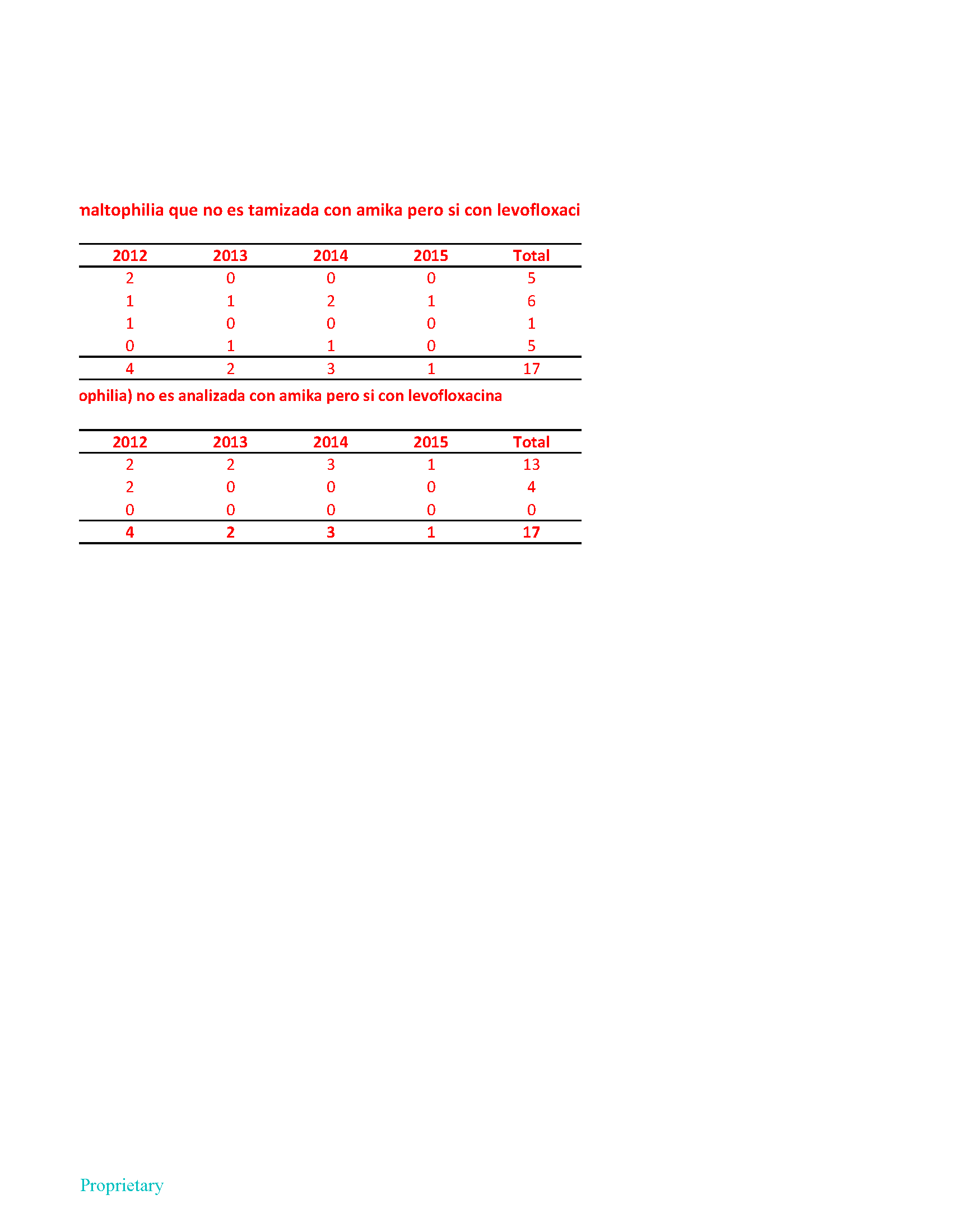

Supplement: S1 Dataset — (ZIP) [file pone.0198621.s005.zip › S1 Data set_Page_3.tiff]
