# Supplementary material for: Antimicrobial susceptibility of gram-negative bacilli isolated from intra-abdominal and urinary-tract infections in Mexico from 2009 to 2015: Results from the Study for Monitoring Antimicrobial Resistance Trends (SMART)
Source: PLoS One. 2018 Jun 21;13(6):e0198621. doi: 10.1371/journal.pone.0198621 (PMC6013120; doi:10.1371/journal.pone.0198621)
Supplement: S1 Tables — Distribution of isolates from intra-abdominal infections, urinary-tract infections by National Institutes of Health and General Hospitals per year, from SMART study in Mexico between 2009 and 2015 (A and B respectively). Antimicrobial susceptibilities of the most common isolates including the ESBL-producing ones for the National Institutes of Health, and the General Hospitals from intra-abdominal infections and urinary-tract infections, from SMART study in Mexico from 2009 to 2015 (C and D respectively). (DOCX) [file pone.0198621.s004.docx]

**Supplementary Information**

**Table A –** Distribution of isolates from intra-abdominal infections by National Institutes of Health and General Hospitals per year, from SMART study in Mexico between 2009 and 2015.

|  |  | **National Institutes of Health** | | | | | | | | **General Hospitals** | | | | | | | |
| --- | --- | --- | --- | --- | --- | --- | --- | --- | --- | --- | --- | --- | --- | --- | --- | --- | --- |
| **IAI Pathogen** | **Overall total** | **2009** | **2010** | **2011** | **2012** | **2013** | **2014** | **2015** | **Total** | **2009** | **2010** | **2011** | **2012** | **2013** | **2014** | **2015** | **Total** |
| *Escherichia coli* | 1465 | 111 | 133 | 121 | 124 | 130 | 118 | 117 | 854 | 82 | 103 | 88 | 114 | 80 | 53 | 91 | 611 |
| *Klebsiella pneumoniae* | 308 | 6 | 9 | 19 | 21 | 21 | 36 | 36 | 148 | 19 | 15 | 17 | 22 | 28 | 30 | 29 | 160 |
| *Pseudomonas aeruginosa* | 261 | 29 | 19 | 15 | 14 | 11 | 14 | 7 | 109 | 31 | 26 | 25 | 14 | 30 | 9 | 17 | 152 |
| *Acinetobacter baumannii* | 178 | 2 | 2 | 3 | 4 | 1 | 1 | 0 | 13 | 22 | 24 | 34 | 22 | 20 | 23 | 20 | 165 |
| *Enterobacter cloacae* | 122 | 10 | 3 | 11 | 7 | 6 | 11 | 3 | 51 | 16 | 12 | 5 | 10 | 9 | 5 | 14 | 71 |
| *Proteus mirabilis* | 45 | 4 | 1 | 1 | 2 | 5 | 4 | 2 | 19 | 2 | 6 | 1 | 3 | 6 | 6 | 2 | 26 |
| *Klebsiella oxytoca* | 54 | 5 | 0 | 3 | 2 | 1 | 3 | 15 | 29 | 3 | 1 | 4 | 0 | 3 | 7 | 7 | 25 |
| *Morganella morganii* | 38 | 4 | 1 | 5 | 3 | 0 | 2 | 3 | 18 | 1 | 1 | 3 | 4 | 6 | 2 | 3 | 20 |
| *Citrobacter freundii* | 42 | 3 | 1 | 5 | 4 | 2 | 5 | 5 | 25 | 1 | 2 | 6 | 2 | 2 | 1 | 3 | 17 |
| *Serratia marcescens* | 24 | 2 | 1 | 0 | 3 | 0 | 2 | 1 | 9 | 4 | 0 | 0 | 0 | 2 | 5 | 4 | 15 |
| *Enterobacter aerogenes* | 26 | 4 | 4 | 2 | 1 | 3 | 3 | 2 | 19 | 2 | 3 | 0 | 0 | 1 | 1 | 0 | 7 |
| *Proteus vulgaris* | 19 | 1 | 0 | 2 | 1 | 0 | 3 | 3 | 10 | 3 | 1 | 1 | 1 | 2 | 0 | 1 | 9 |
| *Stenotrophomonas maltophilia* | 16 | 0 | 0 | 0 | 2 | 1 | 3 | 1 | 7 | 1 | 2 | 3 | 1 | 1 | 1 | 0 | 9 |
| *Others* | 84 | 9 | 6 | 6 | 9 | 5 | 3 | 6 | 44 | 6 | 4 | 4 | 6 | 9 | 4 | 7 | 40 |
| ***Total*** | 2682 | 190 | 180 | 193 | 197 | 186 | 208 | 201 | 1355 | 193 | 200 | 191 | 199 | 199 | 147 | 198 | 1327 |

**Table B –** Distribution of isolates from urinary-tract infections by National Institutes of Health and General Hospitals per year, from SMART study in Mexico between 2009 and 2015.

|  |  | **National Institutes of Health** | | | | | | | **General Hospitals** | | | | | | |
| --- | --- | --- | --- | --- | --- | --- | --- | --- | --- | --- | --- | --- | --- | --- | --- |
| **UTI Pathogen** | **Overall total** | **2010** | **2011** | **2012** | **2013** | **2014** | **2015** | **Total** | **2010** | **2011** | **2012** | **2013** | **2014** | **2015** | **Total** |
| *Escherichia coli* | 790 | 44 | 74 | 80 | 66 | 78 | 41 | 383 | 63 | 66 | 78 | 55 | 90 | 55 | 407 |
| *Klebsiella pneumoniae* | 167 | 16 | 6 | 2 | 12 | 6 | 25 | 67 | 13 | 9 | 9 | 15 | 35 | 19 | 100 |
| *Pseudomonas aeruginosa* | 80 | 20 | 12 | 8 | 9 | 2 | 5 | 56 | 3 | 5 | 4 | 5 | 5 | 2 | 24 |
| *Acinetobacter baumannii* | 26 | 2 | 3 | 0 | 2 | 1 | 0 | 8 | 1 | 8 | 2 | 5 | 1 | 1 | 18 |
| *Enterobacter cloacae* | 25 | 0 | 0 | 1 |  | 0 | 1 | 2 | 4 | 3 | 4 | 0 | 3 | 9 | 23 |
| *Proteus mirabilis* | 44 | 0 | 3 | 3 | 2 | 5 | 10 | 23 | 10 | 1 | 0 | 6 | 2 | 2 | 21 |
| *Klebsiella oxytoca* | 16 | 3 | 1 | 0 | 0 | 0 | 5 | 9 | 1 | 1 | 0 | 3 | 1 | 1 | 7 |
| *Morganella morganii* | 23 | 1 |  | 0 | 0 | 2 | 5 | 8 | 1 | 3 | 1 | 4 | 4 | 2 | 15 |
| *Citrobacter freundii* | 17 | 0 | 1 | 3 | 5 | 0 | 2 | 11 | 0 | 0 | 1 | 1 | 2 | 2 | 6 |
| *Serratia marcescens* | 12 | 1 | 0 | 0 | 1 | 2 | 3 | 7 | 0 | 0 | 0 | 0 | 3 | 2 | 5 |
| *Enterobacter aerogenes* | 7 | 1 | 0 | 0 | 1 | 0 | 0 | 2 | 1 | 0 | 0 | 3 | 1 | 0 | 5 |
| *Proteus vulgaris* | 4 | 0 | 0 | 0 | 0 | 0 | 0 | 0 | 0 | 0 | 0 | 2 | 2 | 0 | 4 |
| *Stenotrophomonas maltophilia* | 4 | 1 | 0 | 1 | 0 | 0 | 0 | 2 | 1 | 0 | 1 | 0 | 0 | 0 | 2 |
| *Others* | 20 | 2 | 0 | 0 | 2 | 0 | 3 | 7 | 3 | 3 | 0 | 1 | 1 | 5 | 13 |
| ***Total*** | 1235 | 91 | 100 | 98 | 100 | 96 | 100 | 585 | 101 | 99 | 100 | 100 | 150 | 100 | 650 |

**Table C** – Antimicrobial susceptibilities of the most common isolates including the ESBL-producing ones for the National Institutes of Health, from intra-abdominal infections and urinary-tract infections, from SMART study in Mexico from 2009 to 2015.

| **Pathogen** |  | **% Susceptibility for National Institutes of Health** | | | | | | | | | | | |
| --- | --- | --- | --- | --- | --- | --- | --- | --- | --- | --- | --- | --- | --- |
|  | **Type of infection ^a)^** | **ETP** | **IMP** | **TZP** | **SAM** | **FOX** | **CAZ** | **CRO** | **CTX** | **FEP** | **LVX** | **CIP** | **AMK** |
| *Escherichia coli* | CA | 97 | 100 | 83 | 19 | 77 | 40 | 35 | 35 | 38 | 24 | 25 | 98 |
|  | N | 100 | 99 | 84 | 21 | 75 | 51 | 44 | 46 | 47 | 31 | 31 | 97 |
| *Escherichia coli, ESBL* | CA | 97 | 100 | 78 | 9 | 76 | 8 | 1 | 1 | 1 | 9 | 9 | 96 |
|  | N | 100 | 100 | 81 | 4 | 66 | 11 | 0 | 1 | 1 | 4 | 4 | 96 |
| *Escherichia coli, non ESBL* | CA | 99 | 99 | 90 | 31 | 71 | 81 | 78 | 78 | 88 | 49 | 50 | 99 |
|  | N | 100 | 99 | 83 | 34 | 82 | 84 | 83 | 84 | 92 | 55 | 55 | 100 |
| *Klebsiella pneumoniae* | CA | 95 | 95 | 84 | 48 | 87 | 63 | 62 | 62 | 62 | 78 | 65 | 99 |
|  | N | 99 | 99 | 86 | 49 | 90 | 70 | 59 | 60 | 62 | 79 | 71 | 99 |
| *Klebsiella pneumoniae, ESBL* | CA | 97 | 100 | 64 | 0 | 89 | 4 | 1 | 1 | 1 | 67 | 20 | 98 |
|  | N | 100 | 100 | 74 | 7 | 96 | 31 | 0 | 7 | 7 | 53 | 32 | 100 |
| *Klebsiella pneumoniae, non ESBL* | CA | 91 | 91 | 91 | 73 | 86 | 91 | 90 | 90 | 91 | 88 | 88 | 96 |
|  | N | 98 | 98 | 97 | 78 | 86 | 94 | 94 | 94 | 98 | 93 | 90 | 99 |
| *Pseudomonas aeruginosa* | CA | 0 | 40 | 47 | 0 | 0 | 48 | 0 | 0 | 50 | 39 | 39 | 64 |
|  | N | 0 | 51 | 45 | 0 | 0 | 40 | 0 | 0 | 48 | 48 | 51 | 58 |
| *Acinetobacter baumannii* | CA | 0 | 31 | 23 | 49 | 0 | 31 | 8 | 15 | 15 | 8 | 0 | 23 |
|  | N | 0 | 0 | 0 | 33 | 0 | 0 | 0 | 0 | 0 | 33 | 33 | 0 |
| *Enterobacter cloacae* | CA | 77 | 98 | 70 | 22 | 0 | 57 | 57 | 54 | 63 | 100 | 87 | 100 |
|  | N | 77 | 100 | 53 | 29 | 7 | 51 | 48 | 48 | 78 | 91 | 90 | 94 |
| *Proteus mirabilis* | CA | 100 | 44 | 100 | 83 | 100 | 100 | 94 | 94 | 100 | 100 | 80 | 100 |
|  | N | 100 | 50 | 90 | 55 | 100 | 90 | 90 | 90 | 80 | 70 | 55 | 100 |
| *Proteus mirabilis, ESBL* | CA | 100 | 0 | 100 | 0 | 100 | 100 | 0 | 0 | 100 | 100 | 0 | 100 |
|  | N | 100 | 0 | 100 | 100 | 100 | 0 | 0 | 0 | 0 | 0 | 0 | 100 |
| *Proteus mirabilis, non ESBL* | CA | 100 | 46 | 100 | 84 | 100 | 100 | 96 | 96 | 100 | 100 | 83 | 100 |
|  | N | 100 | 56 | 89 | 50 | 100 | 100 | 100 | 100 | 89 | 78 | 61 | 100 |
| *Klebsiella oxytoca* | CA | 100 | 100 | 98 | 45 | 89 | 73 | 65 | 65 | 73 | 59 | 59 | 100 |
|  | N | 100 | 88 | 69 | 53 | 88 | 63 | 54 | 61 | 73 | 61 | 61 | 100 |
| *Klebsiella oxytoca, ESBL* | CA | 100 | 100 | 100 | 0 | 100 | 17 | 0 | 0 | 17 | 17 | 17 | 100 |
|  | N | 100 | 100 | 67 | 0 | 100 | 33 | 0 | 0 | 0 | 0 | 0 | 100 |
| *Klebsiella oxytoca, non ESBL* | CA | 100 | 100 | 98 | 65 | 85 | 98 | 98 | 98 | 98 | 80 | 80 | 100 |
|  | N | 100 | 83 | 75 | 72 | 83 | 83 | 75 | 83 | 100 | 83 | 83 | 100 |

ETP: ertapenem, IMP: imipenem, TZP: Piperacillin-Sulbactam, SAM: Ampicillin-Sulbactam, FOX: cefoxitin, CAZ: ceftazidime, CRO: ceftriaxone, CTX: cefotaxime, FEP: cefepime, LVX: levofloxacin, CIP: ciprofloxacin and AMK: amikacin. ^a)^ The infections were categorized as community-acquired (CA) and nosocomial (N) defined, respectively, as isolates obtained in <48 hours or >48h after hospitalization; These MIC breakpoints have not been defined by the Clinical and Laboratory Standards Institute.

**Table D** – Antimicrobial susceptibilities of the most common isolates including the ESBL-producing ones for the General Hospitals, from intra-abdominal infections and urinary-tract infections, from SMART study in Mexico from 2009 to 2015.

| **Pathogen** |  | **% Susceptibility for General Hospitals** | | | | | | | | | | | |
| --- | --- | --- | --- | --- | --- | --- | --- | --- | --- | --- | --- | --- | --- |
|  | **Type of infection ^a)^** | **ETP** | **IMP** | **TZP** | **SAM** | **FOX** | **CAZ** | **CRO** | **CTX** | **FEP** | **LVX** | **CIP** | **AMK** |
| *Escherichia coli* | CA | 100 | 99 | 88 | 29 | 81 | 52 | 49 | 48 | 52 | 43 | 41 | 96 |
|  | N | 99 | 100 | 88 | 21 | 81 | 53 | 49 | 48 | 51 | 41 | 40 | 97 |
| *Escherichia coli, ESBL* | CA | 99 | 98 | 82 | 9 | 73 | 5 | 0 | 0 | 1 | 21 | 19 | 92 |
|  | N | 99 | 100 | 81 | 5 | 71 | 11 | 1 | 1 | 2 | 11 | 10 | 93 |
| *Escherichia coli, non ESBL* | CA | 100 | 100 | 94 | 48 | 91 | 95 | 96 | 95 | 100 | 64 | 61 | 99 |
|  | N | 100 | 100 | 94 | 36 | 90 | 95 | 95 | 95 | 99 | 69 | 67 | 99 |
| *Klebsiella pneumoniae* | CA | 100 | 100 | 84 | 49 | 85 | 57 | 54 | 55 | 56 | 84 | 61 | 96 |
|  | N | 97 | 98 | 85 | 47 | 88 | 62 | 53 | 55 | 60 | 79 | 63 | 95 |
| *Klebsiella pneumoniae, ESBL* | CA | 100 | 100 | 72 | 8 | 82 | 5 | 0 | 0 | 3 | 69 | 22 | 91 |
|  | N | 97 | 100 | 70 | 7 | 85 | 15 | 0 | 0 | 12 | 62 | 28 | 92 |
| *Klebsiella pneumoniae, non ESBL* | CA | 100 | 100 | 95 | 84 | 93 | 100 | 99 | 100 | 100 | 95 | 95 | 100 |
|  | N | 98 | 97 | 95 | 78 | 90 | 96 | 90 | 93 | 98 | 94 | 89 | 98 |
| *Pseudomonas aeruginosa* | CA | 0 | 78 | 66 | 0 | 0 | 71 | 0 | 0 | 71 | 64 | 64 | 75 |
|  | N | 0 | 72 | 73 | 0 | 0 | 69 | 0 | 0 | 70 | 71 | 73 | 77 |
| *Acinetobacter baumannii* | CA | 0 | 36 | 18 | 35 | 0 | 9 | 3 | 3 | 12 | 9 | 3 | 32 |
|  | N | 0 | 20 | 7 | 17 | 0 | 7 | 7 | 6 | 7 | 8 | 7 | 16 |
| *Enterobacter cloacae* | CA | 87 | 100 | 70 | 13 | 0 | 47 | 47 | 53 | 63 | 87 | 83 | 93 |
|  | N | 88 | 96 | 68 | 28 | 8 | 59 | 52 | 53 | 66 | 86 | 83 | 91 |
| *Proteus mirabilis* | CA | 100 | 30 | 100 | 90 | 100 | 100 | 90 | 100 | 100 | 90 | 80 | 100 |
|  | N | 100 | 24 | 99 | 78 | 95 | 99 | 91 | 97 | 95 | 89 | 69 | 100 |
| *Proteus mirabilis, ESBL* | CA | - | - | - | - | - | - | - | - | - | - | - | - |
|  | N | - | - | - | - | - | - | - | - | - | - | - | - |
| *Proteus mirabilis, non ESBL* | CA | 100 | 30 | 100 | 90 | 100 | 100 | 90 | 100 | 100 | 90 | 80 | 100 |
|  | N | 100 | 24 | 99 | 78 | 95 | 99 | 91 | 97 | 95 | 89 | 69 | 100 |
| *Klebsiella oxytoca* | CA | 100 | 100 | 96 | 68 | 96 | 96 | 96 | 96 | 96 | 82 | 82 | 100 |
|  | N | 100 | 94 | 91 | 62 | 97 | 91 | 82 | 85 | 85 | 88 | 85 | 100 |
| *Klebsiella oxytoca, ESBL* | CA | 100 | 100 | 0 | 0 | 0 | 0 | 0 | 0 | 0 | 0 | 0 | 100 |
|  | N | 100 | 100 | 33 | 0 | 67 | 33 | 0 | 0 | 0 | 67 | 33 | 100 |
| *Klebsiella oxytoca, non ESBL* | CA | 100 | 100 | 100 | 71 | 100 | 100 | 100 | 100 | 100 | 86 | 86 | 100 |
|  | N | 100 | 93 | 100 | 73 | 100 | 100 | 97 | 100 | 100 | 93 | 93 | 100 |

ETP: ertapenem, IMP: imipenem, TZP: Piperacillin-Sulbactam, SAM: Ampicillin-Sulbactam, FOX: cefoxitin, CAZ: ceftazidime, CRO: ceftriaxone, CTX: cefotaxime, FEP: cefepime, LVX: levofloxacin, CIP: ciprofloxacin and AMK: amikacin. ^a)^ The infections were categorized as community-acquired (CA) and nosocomial (N) defined, respectively, as isolates obtained in <48 hours or >48h after hospitalization; These MIC breakpoints have not been defined by the Clinical and Laboratory Standards Institute.
